# Soluble Collagen VI treatment enhances mesenchymal stem cells expansion for engineering cartilage

**DOI:** 10.1002/btm2.10078

**Published:** 2017-09-21

**Authors:** Piera Smeriglio, Jieun Lee, Nidhi Bhutani

**Affiliations:** ^1^ Dept. of Orthopaedic Surgery Stanford University School of Medicine Stanford CA 94305

**Keywords:** cell expansion, Col VI, mesenchymal stem cells

## Abstract

Bone Marrow‐derived mesenchymal stem cells (BM‐MSC) are an attractive source for cell‐based therapies in cartilage injury owing to their efficient differentiation into chondrocytes and their immune‐suppressive abilities. However, their clinical use is hampered by a scarcity of cells leading to compromised efficacy. While expansion of human MSC ex vivo can potentially overcome the scarcity of cells, current methods lead to a rapid loss of the stem cell properties. In this study, we report soluble Collagen VI (cartilage pericellular matrix component) as a potential biologic that can expand the MSC population while maintaining the stem cell phenotype as confirmed by expression of the stem cell markers CD105 and CD90. Short‐term treatment with Collagen VI additionally retains the potential of MSC to differentiate into mature chondrocytes in pellet culture. Cartilage pellets generated from MSC treated with Collagen VI or control express comparable amounts of the chondrogenic markers Collagen II, Aggrecan and Sox9, and the extracellular glycosaminoglycans. Our observations confirm that the use of the endogenous and cartilage‐specific factor Collagen VI is valuable for a rapid and efficient expansion of MSC for potential use in cartilage regeneration and osteoarthritis.

## 1 INTRODUCTION

Osteoarthritis (OA) is a multifactorial disease that affects the articular cartilage causing deterioration of the joint function. Cartilage injuries and trauma are difficult to repair even in young adults due to the poor regenerative potential of the cartilage tissue, and greatly accelerates OA development.^1^ Current treatments of OA are mainly palliative and aim at pain and symptoms management rather than disease modification.^2^ Lack of any disease modifying OA drugs (DMOADs) calls for new and effective therapies to repair and regenerate damaged articular cartilage, and to restore joint homeostasis to delay OA progression.

Current cell‐based clinical therapies for cartilage repair include the use of autologous chondrocytes or autologous cells from mesenchymal tissues. Stimulation of the endogenous stem cell populations to repair cartilage injuries through microfracture leads to inefficient regeneration and formation of fibrocartilage tissue rather than hyaline cartilage.^3^ Autologous articular chondrocyte implantation (ACI) has been clinically approved but the applications have been limited by a paucity of cells and production of functionally inferior fibrocartilage. Mesenchymal stem cells (MSCs) and adipose‐derived stem cells (ADSCs) have been investigated to engineer and repair cartilage in basic and early translational studies. MSCs are used for treatment of multiple diseases for their ability to differentiate toward multiple cell types including chondrocytes, bone, and adipocytes, combined to their capacity to secrete anti‐inflammatory factors that mitigate inflammation.^4^ The role of inflammation in the development of OA is being increasingly recognized.^5^ As such, the beneficial anti‐inflammatory effects of MSC in prevention or delay of the onset of OA is of substantial interest. Currently, MSCs are being explored as a treatment modality for multiple disease states in hundreds of Phase I and II clinical trials.^6^ A major limitation to clinical translation has been the scarcity of these adult stem cells. While expansion of MSCs ex vivo can solve the scarcity of the cell source, in vitro stem cell expansion frequently leads to a rapid loss of the stem cell properties and potency. Multiple studies have been devoted to overcome this limitation and different signaling pathways like the FGF or Wnt can be modulated to maintain the stem cell characteristics of MSCs.^7^, ^8^, ^9^ Since these signaling pathways can have multiple effects on tissues other than cartilage, there is a high clinical relevance for the identification and development of biologics that are intrinsic to the cartilage tissue and hence can be more specific.

It is widely accepted that both the extracellular matrix (ECM) and the pericellular matrix (PCM) play a critical role in cartilage function especially for maintaining its biochemical and biomechanical properties.^10^ Our recent studies have demonstrated ECM proteins to be a major differential between juvenile and adult cartilage suggesting that the ECM interactions with the chondrocytes also regulate the regenerative capacity of cartilage.^11^ Therefore, the emerging understanding of the crosstalk between chondrocytes and ECM or PCM components is required and should be taken in consideration for cartilage tissue engineering. Collagen VI (Col VI) is a major constituent of the chondrocyte PCM consisting of three major α‐chains, α1, α2, and α3, along with alternate subunits α4, α5, or α6 that can substitute for α3.^12^, ^13^ We have previously demonstrated that short‐term treatment of human chondrocytes with soluble Collagen VI (Col VI) and not Collagen I, can increase their number without adversely affecting their ability to generate cartilage.^14^ As an extension of that study, we aimed to use Col VI for the expansion of the MSC population preserving their stem cell properties and maintaining their potential to efficiently differentiate into cartilage.

## 2 METHODS

### 2.1 hMSCs culture

Human MSCs (NHAC‐kn) from two adult donors (19 and 30 years old, both males) were purchased from Lonza (Clonetics™, Lonza Walkersville Inc., Walkersville, MD) and cultured in monolayer for limited passages (between passages 2 and 4) using Mesenchymal Stem Cell Growth Medium (MSCGM™, Lonza, Walkersville, MD) as per manufacturer instructions. Cells were detached by adding a sufficient volume of Lonza Trypsin/EDTA (CC‐3232) solution to cover the cell layer (approx. 0.05 ml/cm) and centrifuged 150 × *g* for 5 min at room temperature. Pellet formation was induced by centrifugation of 1 million cells per pellet in a 15 ml conic tube at 150 × *g* for 5 min at room temperature as per manufacturer instruction. Tube cap was loosen to allow gas exchange and the tubes incubated at 37°C, in a humidified atmosphere of 5% CO_2_. After 24 hr, chondrogenic differentiation of the pellets was induced in Chondrocyte Differentiation Medium (CDM™, Lonza, Walkersville, MD) and pellets were maintained in culture for 21 days. 0.5 ml of freshly prepared complete chondrogenic medium was replaced every 2 days by complete aspiration of the medium carefully avoiding the pellet.

### 2.2 Collagen VI treatment and cell growth

Collagen VI (BD 354261) (BD Biosciences, San Jose, CA) was dissolved in a 1.25 mM Tris solution. hMSCs were plated in monolayers at 2,000 cells per well in duplicates in 96‐well plates and cultured for 24 hr in complete medium. After 24 hr, cells were treated with control Tris or medium containing 2.5 μg/ml of recombinant human Collagen VI, with media and recombinant protein replacement every day for 4 days. Cell viability was assayed daily with the PrestoBlue Cell Viability Reagent kit (Life Technologies, Carlsbad, CA) as previously described.^14^ Fluorescence intensity of the PrestoBlue reagent reduced by living cells was measured at 690nm (650nm excitation wavelength) with a microplate reader (Molecular Devices, Sunnyvale, CA).

### 2.3 Flow cytometry

Cells were lifted using TrypLE Express Enzyme solution (Life Technologies, Carlsbad, CA), fixed in BD Cytofix buffer (BD Biosciences, San Jose, CA) for 20 min at room temperature and permeabilized with BD Permeabilization/Wash buffer (BD Biosciences, San Jose, CA) at 1 × 10^7^ cells per 1 ml for 10 min. Cells were stained with the following primary antibodies: mouse anti‐human Sox9 (Abcam, Cambridge, MA; 76997, 1:200), mouse anti‐human CD44‐PE/Cy7 (Abcam 46793, 1:100), mouse anti‐human CD90‐FITC and mouse CD105‐Alexa 647 (both from R&D, Minneapolis, MN; FMC002), for 30 min at the concentration suggested by the manufacturer. Secondary antibody (donkey anti‐mouse IgG Alexa 488, Invitrogen, Carlsband, CA) was diluted 1:250. Cells were scanned using a LSR II flow cytometer and analyzed with Flowjo software.

### 2.4 Gene expression analyses

For the monolayer cultures, total RNA was extracted using the RNeasy mini kit (Qiagen, Valencia, CA) and for pellet cultures, total RNA was obtained using TRIzol (Invitrogen, Carlsbad, CA). RNA from each sample was reversed transcribed using the High Capacity cDNA Reverse Transcription Kit (Applied Biosystems, Foster City, CA) and quantitative PCR was performed using TaqMan gene‐specific expression arrays for Type II collagen—chain alpha 1 (*Col2a1*) (Hs00264051_m1), *Sox9* (Hs00165814_m1), Aggrecan (*Acan*) (Hs00153936_m1), and metalloproteinase 13 (*MMP13*) (Hs00942584_m1) with a universal mastermix (Applied Biosystems, Foster City, CA). The following cycling protocol was used: 2 min 50°C, 10 min 95°C, 40 cycles of 15 s 95°C and 1 min 60°C. Gene expression levels were normalized internally to *GAPDH*. Changes in expression were calculated from threshold cycle (Ct) values, where relative gene expression was 2^−ΔΔCt15^.

### 2.5 Immunohistochemistry

hMSC pellets were fixed in 4% paraformaldehyde (Sigma, St Louis, MO) and embedded in paraffin. Pellet sections (10 μm) were permeabilized in cold methanol (Sigma, St Louis, MO), before blocking in PBS containing 1% BSA, 10% FBS, and 0.4% Triton X‐100 and then incubated with the following primary antibodies overnight: rabbit polyclonal Col2a1 (Abcam, Cambridge, MA 34712; 1:100), Aggrecan (1:500), rabbit polyclonal Col6a1 (Santa Cruz, Santa Cruz, CA 20649, 1:100) at the specified dilutions. The antibody anti‐Aggrecan used for this study was a kind gift from Prof. R. L. Smith.^16^ The following day, cells were washed in PBS and incubated for 1 hr in secondary antibody (Alexa 594 goat anti‐rabbit 1:250, Invitrogen, Carlsband, CA) and cellular DNA was counterstained with DAPI (Life Technologies, Carlsband, CA).

### 2.6 Biochemical analyses

DNA and sulfated glycosaminoglycan (sGAG) production were quantified. DNA content was measured using the PicoGreen assay (Molecular Probes, Eugene, OR) with Lambda phage DNA as standard. sGAG content was quantified using the 1,9‐dimethylmethylene blue (DMMB) dye‐binding assay with shark chondroitin sulfate (Sigma, St. Louis, MO) as standard.

### 2.7 Statistical analyses

Data are reported as mean ± standard deviation (SD). Statistical significance of data was determined by applying a two‐tailed Student's *t* test and *p* values less than .05 are reported as significant.

## 3 RESULTS

### 3.1 Collagen VI (Col VI) treatment enhances hMSC proliferation

**Figure 1:**
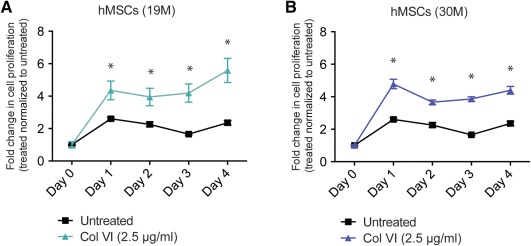
Col VI treatment enhances hMSC proliferation. hMSC from 19‐year‐old male donor (A) and 30‐year‐old male donor (B) upon treatment with soluble Col VI (2.5 μg/ml) in monolayer cultures for 4 days. COL VI was replenished daily. Fold increase in cell number is indicated relative to day 0 and normalized to the control at each time point. Data represent three biological replicates and are expressed as mean ± SD. *Statistical significance where *p* < .05

In the present study, we aimed to study the direct effect of soluble Col VI on human bone marrow‐derived MSCs (hMSC) fate and function. A quantitative fluorescence‐based assay reflecting the metabolic activity of live cells (Prestoblue, Life technologies) was used to measure cell growth, as described previously.^14^ In this assay, the reagent resazurin is nonfluorescent but is converted to fluorescent resorufin in the reducing environment of living cells, allowing a quantitative measurement of relative cell numbers. The advantage of this reagent is both high sensitivity allowing detection of relatively low cell numbers reproducibly as well as minimal toxicity such that the reagent can be used at regular intervals during continuous cell culture. As shown in Figure 1, we observed a significant increase in cell proliferation in hMSC from two different healthy donors (19 and 30 years old, both males) upon treatment with recombinant Col VI (2.5 μg/ml) for 4 days compared to vehicle treated controls. The hMSC were plated at a cell number of 2,000 and their cell growth was monitored every day in the absence or presence of Col VI with the fluorescence assay for 4 days. Soluble Col VI (2.5 μg/ml) was replenished daily. We observed a significant twofold to threefold increase in the number of both hMSC samples upon treatment.

### 3.2 Col VI treatment maintains MSC phenotype

**Figure 2:**
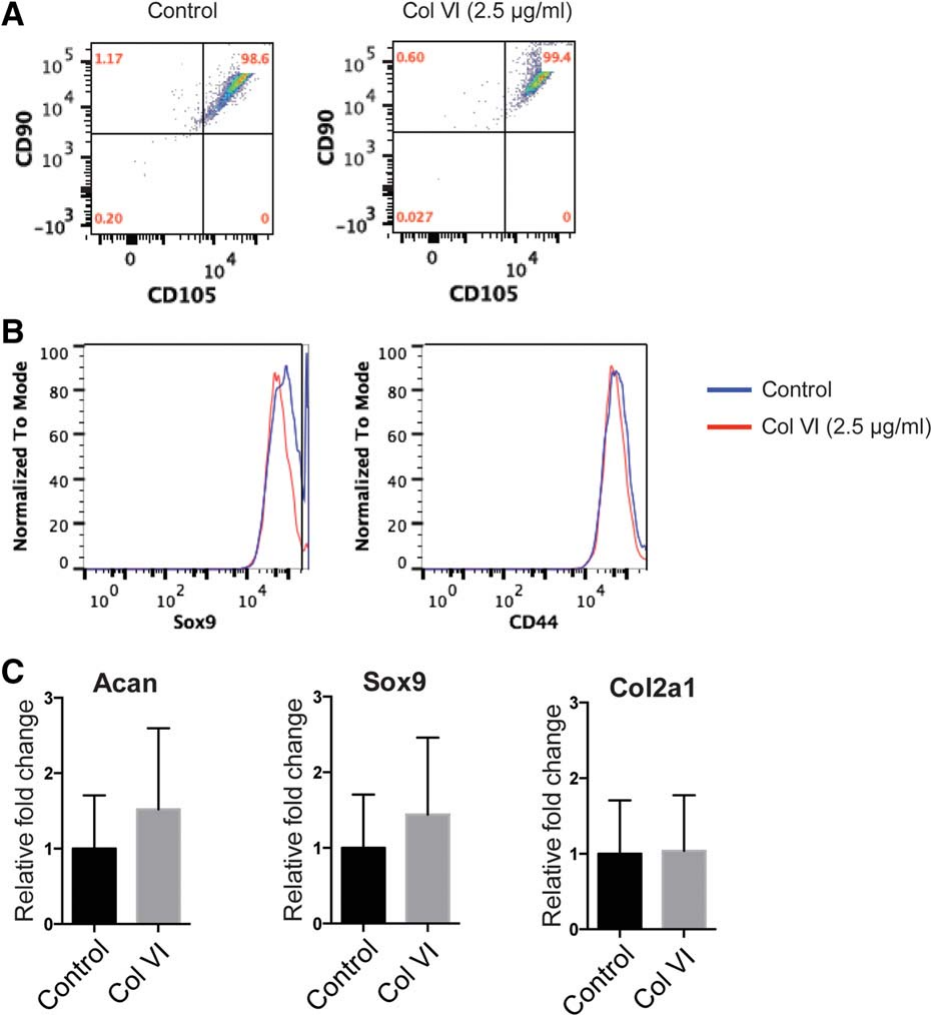
Col VI treatment maintains mesenchymal stem cell phenotype. (A) FACS analysis for hMSC stemness markers CD90 (Thy‐1) and CD105 (Endoglin) expression in hMSC treated with control or 2.5 μg/ml Col VI for 2 days. (B) FACS analyses of chondrogenic markers SOX9 and CD44 expression in hMSC treated with control or 2.5 μg/ml Col VI for 2 days. (C) Gene expression for chondrocyte markers Acan, Sox9, and Col2a1 was assayed in hMSC after treatment with control or 2.5 μg/ml Col VI for 2 days

A fundamental issue for culture and expansion of MSCs and their use in regenerative medicine approaches is their tendency to rapidly lose their stem cell characteristics upon culture. In order to test if the treatment with soluble Collagen VI maintains hMSC phenotype, we analyzed the expression of the characteristic stem cell markers on the cell surface of hMSC, CD105, and CD90 by fluorescence activated cell sorting (FACS) (Figure 2A). We observed that Col VI‐treated cell populations were similar to untreated populations with both retaining high expression of CD90 and CD105 after 2 days of Col VI treatment. It is to be noted that at a single cell level, 99% of hMSC were double positive for CD90 and CD105 both before and after Col VI treatment. Furthermore, Col VI treatment did not alter the expression of the chondrogenic transcription factor Sox9 (low expression in MSC) or increase the cartilage surface marker CD44 in hMSC as assayed by FACS before and after 2 days of treatment (Figure 2B). Similar trend was observed at gene expression level where the expression of chondrogenic markers Sox9, Acan, and Col2a1 was not induced upon Col VI treatment (Figure 2C) suggesting that Col VI treatment did not skew the hMSC toward a chondrogenic lineage.

### 3.3 Col VI treatment retains the chondrogenic differentiation potential of hMSC

**Figure 3:**
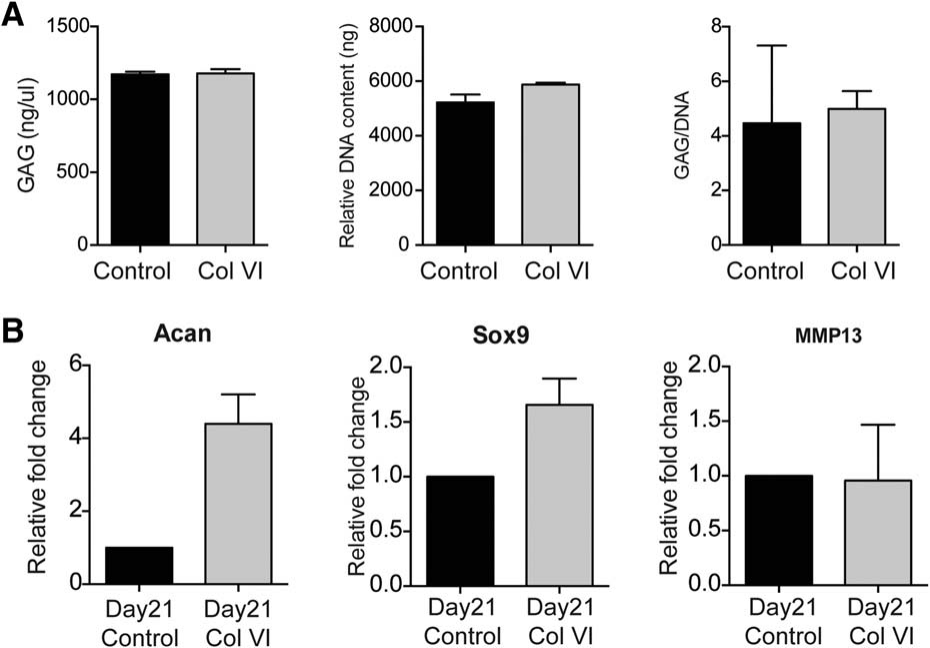
Col VI treatment retains the chondrogenic differentiation potential of hMSC. (A) Glycosaminoglycan (GAG) content, DNA content, and GAG normalized to DNA content in pellets seeded with control and COL VI‐treated hMSC after 21 days of culture. (B) Gene expression levels for chondrocyte markers Acan, Sox9, and MMP13 were assayed in hMSC pretreated with COL VI and cultured for 21 days

**Figure 4:**
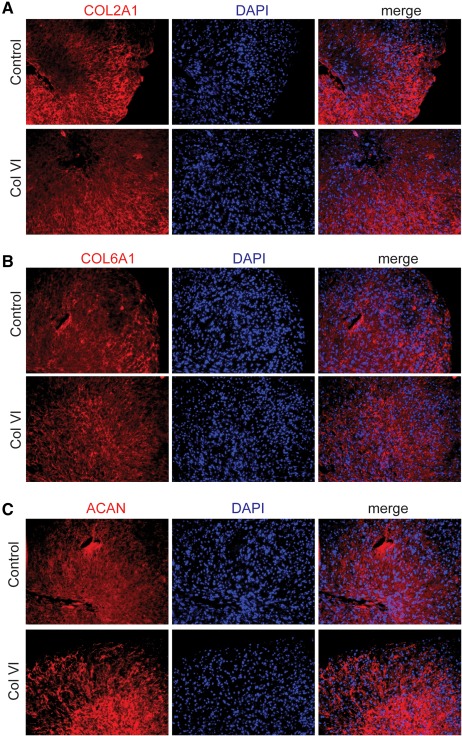
COL VI‐treated hMSC show efficient cartilage differentiation similar to control hMSC. Chondrogenic marker expression in cartilage pellets after 21 days of culture for control or Col VI‐treated hMSC. Red: immunofluorescence staining for (A) Collagen 2A1 (COL2A1), (B) Collagen 6A1 (COL6A1), and (C) Aggrecan (AGC); Blue: cell nuclei stained with DAPI

To assess if the Col VI treatment retains the potential of hMSC to differentiate into mature chondrocytes and engineer cartilage, we performed a three‐dimensional pellet culture of chondrocytes after control or Col VI treatment (2.5 μg/ml) for 2 days. MSCs were treated in monolayer culture, then centrifuged to allow pellet formation (500,000 cells per pellet) and cultured in Transforming growth factor‐beta enriched media (0.01 μg/ml) for 21 days. At the end of the chondrogenic differentiation, pellets were analyzed for chondrogenic gene and protein expression and biochemical properties. We first observed that the DNA content in pellets of cells previously exposed to Col VI was not significantly higher than untreated control at the end of differentiation demonstrating that the proliferative effect is reversible and not sustained over time in the absence of the soluble Col VI (Figure 3A). Furthermore, the glycosaminoglycans (GAG) content quantification showed full maturation of cell pellets in both Col VI treatment and untreated control cells (Figure 3A) suggesting that the chondrogenic potential of hMSC is retained upon Col VI treatment. Additionally, the gene expression of chondrogenic genes like Sox9 and Aggrecan was increased upon differentiation in Col VI‐treated cells. As observed previously, no increase in chondrogenic markers was observed in Col VI‐treated and undifferentiated hMSC, however the prior Col VI treatment appears beneficial to the chondrogenic differentiation of MSCs (Figure 3B). MMP13 gene expression was unchanged by Col VI treatment (Figure 3B). Histological evaluation and immunostaining of the cell pellets after 21 days of three‐dimensional pellet culture showed similar level of expression for cartilage‐specific Col2a1 and Aggrecan, confirming that Col VI‐treated hMSC attained a mature chondrogenic phenotype similar to the control cells. Col I and Col X expression remained minimal (data not shown). Staining for endogenous Col VI was similar for the control and Col VI‐treated hMSC showing the absence of any feedback regulation of the soluble Col VI on the endogenous Col6a1 locus (Figure 4).

## 4 DISCUSSION

Although the benefits of MSC in cartilage tissue engineering have been investigated for many years, more recent studies highlight the benefits of MSC as a “secretory” reserve of anti‐inflammatory factors in addition to their ability to differentiate into cartilage.^17^ The characteristics that make MSC an attractive candidate for knee OA are their safety and lack of immune rejection in the secluded knee joint. Positive outcomes for pain management and function improvement after MSC injection into osteoarthritic knee joints have been reported in a few clinical trails^18^, ^19^, ^20^ although more detailed analyses of the long‐term effects need to be performed. The barriers to the clinical translation for the use of MSC are manifold: the low frequency of MSC in human bone‐marrow, lack of effective methods for their expansion into sufficient cell numbers, and for ensuring cellular viability after transplantation. The goal of this work is to expand the MSC population with a minimally manipulative technique, herein the short treatment, and preserving the stem cell properties. The treatment with Col VI represents a novel and promising method to expand MSC with the advantage of Col VI being an endogenous component of the cartilage ECM. Therefore, we expect that adverse effects in the native tissue will be minimal.

Col VI is the predominant component of the chondrocytes PCM visualized to be a transducer of signals between the cells and the surrounding ECM.^21^ Here we show that short‐term treatment (48 hr) of MSC with soluble Col VI ensures that the stem cell markers CD90 and CD105 are maintained as shown by the high expression of the surface markers prior and after Col VI treatment. These observations suggest that Col VI treatment would potentially be beneficial for MSC‐based treatments in a variety of diseases. Additionally, both control and Col VI‐treated MSC showed robust cartilage tissue generation when seeded in 3D pellets and cultured in chondrogenic conditions for 3 weeks. The DNA content at day 0 and 21 of differentiation is comparable in both treated and untreated control MSC showing that the proliferative effect induced by soluble Col VI is short‐lived and does not modify the behavior of the cells permanently. Our data demonstrates that the cells previously expanded in 2D upon treatment with soluble Col VI, can differentiate into mature cartilage thus showing that the treatment does not alter their differentiation potential. Expression of chondrogenic markers like Acan, Sox9, Col2a1, and Col6a1 was maintained or enhanced after 3 weeks of culture in Col VI‐treated MSC. Similarly, all the cartilage pellets showed robust COL II and ACAN protein expression as well as high expression for SOX9 and COL VI while dedifferentiation marker COL I and hyperthrophic marker COL X expression remained minimal. An effective chondrogenic differentiation is confirmed by the ability of the expanded cells to deposit an equivalent amount of total GAGs than the untreated control cells. Therefore, the Col VI‐expanded MSC can effectively engineer cartilage tissue, comparable to MSC that have not been treated with Col VI but with the advantage of being higher in number. Although the precise mechanism through which soluble Col VI enhances MSC proliferation is not clear, it has been shown previously that the skeletal muscle stem cells self‐renewal is profoundly reduced in Col6^−/−^ mutant mice.^22^ Engraftment of wild‐type Col6^+/+^ fibroblasts in the mutant mice muscles was able to rescue the defect in stem cell self‐renewal leading the authors to conclude that a modulation of the biomechanical properties of the muscle tissue influenced the stem cell self‐renewal. In a similar fashion, absence of Col VI in Col6^−/−^ mice affects the biomechanical properties of chondrocytes leading to altered chondrocyte behavior and a spontaneous development of hip OA.^21^ We therefore speculate that Col VI may modulate the cell stem proliferation through interacting with surface receptors that likely sense the biomechanical properties of the microenvironment. The identity of these receptors and the downstream signaling pathways will be further investigated in future studies.

In conclusion, in this study we have described a useful and effective method for MSC expansion utilizing a cartilage‐specific factor, Col VI. We envision that Col VI and similar biologics are promising candidates to enhance the effectiveness of MSC‐based therapeutic approaches.

## CONFLICT OF INTEREST

The authors declare no competing interests.
